# Effect of Oral and Prosthodontic Rehabilitation on Nutrition and General Health Outcomes in Edentulous Elderly Populations: A Systematic Review

**DOI:** 10.1155/ijod/5786191

**Published:** 2026-02-12

**Authors:** Anupama Prasad D., Krishna Prasad D., Harshitha Alva, Archana Shetty, Kishore A. Chougule, Hashim Yaacob, Ramya Shenoy

**Affiliations:** ^1^ Department of Prosthodontics, AB Shetty Memorial Institute of Dental Sciences, NITTE (Deemed to be University), Deralakatte, Mangaluru, Karnataka, 575018, India, nitte.edu.in; ^2^ Department of Prosthodontics, Dayananda Sagar College of Dental Sciences, Shavigemalleshwara Hills, Kumaraswamy Layout, Bengaluru, Karnataka, 560111, India, dayanandasagar.edu; ^3^ Department of Prosthodontics, VS Dental College and Hospital, Bengaluru, Karnataka, 560004, India; ^4^ Department of Orthodontics, Tatyasaheb Kore Dental College and Research Centre, Mahatma Gandhi Hospital Campus, Hatkanangale Taluk, Kolhapur District, New Pargaon, Maharashtra, 416113, India, tkdc.org; ^5^ Department of Oral Diagnostic Sciences, Faculty of Dentistry, SEGi University, Kota Damansara, Petaling Jaya, Selangor, Malaysia, segi.edu.my

**Keywords:** complete denture, edentulism, elderly, implant-supported prostheses, nutrition, quality of life

## Abstract

**Introduction:**

Edentulism impairs mastication and dietary choices in older adults, often leading to malnutrition and associated health risks. Despite being a routine practice, the impact of prosthodontic rehabilitation on systemic and nutritional health outcomes is yet unknown.

**Objective:**

To systematically review the effects of prosthodontic rehabilitation on nutritional and general health outcomes in elderly edentulous individuals and compare outcomes across prosthetic modalities.

**Methods:**

A systematic review was conducted per PRISMA 2020 and PROSPERO registration (CRD420251147518) across PubMed, MEDLINE, Cochrane CENTRAL, and Scopus. Eligible studies included randomized clinical trials (RCTs) and clinical and observational studies assessing prosthodontic rehabilitation in adults ≥ 60 years of age that reported nutritional and general health outcomes. The risk of bias was assessed via the risk of bias 2 (RoB 2) for RCTs and the Newcastle–Ottawa Scale (NOS) for observational studies. Narrative synthesis was undertaken as the included studies differed substantially in their designs, interventions, and outcome measures.

**Results:**

Fifteen studies (six RCTs and nine observational) were included. Masticatory function and quality of life related to oral health were consistently enhanced by prosthodontic rehabilitation. When denture provision was coupled with dietary advice, nutritional status improved. Bite power, mastication, and meal variety were all enhanced by implant‐supported and single‐implant overdentures; however, biochemical marker alterations were not consistent. Data pertaining to systemic outcomes are scarce (frailty, morbidity, and mortality).

**Conclusion:**

Prosthodontic rehabilitation in elderly individuals improves chewing and quality of life, with modest but consistent benefits for nutritional outcomes. Stronger effects occur when combined with dietary counseling. Evidence for systemic outcomes remains limited. Future multicenter RCTs with longer follow‐up periods and standardized nutritional endpoints are warranted.

## 1. Introduction

Edentulism is a major oral health condition with profound functional, nutritional, psychological, and systemic implications. It represents the final stage of oral disease progression resulting from cumulative caries, periodontal disease, and other oral pathologies. There are two types of edentulism, namely, partial edentulism and complete edentulism. Partial edentulism is the loss of one or more natural teeth, and complete edentulism is the loss of all natural teeth [1, 2]. Edentulism is one of the most common chronic conditions affecting older adults worldwide. According to estimates from the Global Burden of Disease (GBD) Study, ~353 million people were fully edentulous in 2021, underscoring its status as one of the most debilitating oral conditions globally [3, 4]. Data from the World Health Organization (WHO) indicate that complete tooth loss affects nearly 7% of adults aged 20 years and older, and its prevalence increases sharply to around 23% among those aged 60 years and above [5]. In India, the burden is particularly notable: A recent meta‐analysis reported that 34.6% of adults have experienced tooth mortality, with complete edentulism affecting 10.7% and partial edentulism affecting nearly 58.8% of the population [6].

The consequences of edentulism extend far beyond oral discomfort or appearance. The loss of occluding tooth units significantly impairs mastication, limiting the ability to chew and process a variety of foods. As a result, older adults frequently shift towards softer, carbohydrate‐rich diets and avoid harder, fiber‐ and protein‐rich foods such as fruits, vegetables, legumes, and meats. These dietary changes contribute to nutritional deficiencies, including inadequate intake of protein, fiber, essential fatty acids, and micronutrients such as vitamins A, C, D, B12, iron, and calcium. Such deficiencies not only compromise general health but also exacerbate age‐related conditions like sarcopenia, frailty, decreased immune resilience, delayed tissue repair, and impaired physical functioning. Ultimately, poor nutrition in older adults is associated with increased susceptibility to infections, reduced quality of life, higher rates of hospitalization, and increased mortality [7–9].

The cornerstone of edentulism treatment is prosthodontic rehabilitation, which includes fixed prostheses, implant‐supported overdentures, removable partial dentures (RPDs), and complete dentures (CDs). Following rehabilitation, there is ample evidence of improvements in chewing efficiency and oral health‐related quality of life (OHRQoL) [10, 11]. However, despite improvements in oral function, the extent to which prosthodontic rehabilitation translates into tangible improvements in nutritional status and systemic health remains unclear. Nutritional outcomes include multiple variables—dietary intake, macronutrient and micronutrient adequacy, anthropometric measures such as weight and body mass index (BMI), and biochemical markers such as serum albumin, total protein, hemoglobin, calcium, and lipid profiles. Some randomized controlled trials (RCTs) and longitudinal studies report modest improvements in BMI, Mini Nutritional Assessment (MNA) scores, and dietary variety following prosthodontic rehabilitation, especially when accompanied by personalized dietary counseling [11–13]. Yet, the evidence remains inconsistent. Several studies report negligible or no changes in biochemical markers or systemic measures, highlighting the complex interplay between diet, absorption, metabolism, comorbidities, and prosthetic adaptation [14, 15]. Even when functional outcomes improve significantly, older adults may not modify long‐standing dietary habits, limiting the impact on nutritional biomarkers. Moreover, systemic outcomes such as frailty, hospitalization risk, morbidity, and mortality are rarely assessed, and when reported, sample sizes are often insufficient to determine long‐term effects [16, 17].

Considerable heterogeneity in study designs, prosthetic modalities, outcome measures, and follow‐up durations further complicates efforts to synthesize existing evidence and draw definitive conclusions. Despite the growing literature, there is a lack of consolidated, high‐quality evidence evaluating the broader nutritional and general health effects of prosthodontic rehabilitation in elderly populations. Previous studies have primarily emphasized functional outcomes and quality of life [10, 11], with limited focus on dietary behavior, nutrient adequacy, systemic biomarkers, frailty status, or morbidity. Given the aging global population and the increasing prevalence of edentulism, an integrated understanding of how prosthodontic rehabilitation influences both nutritional and systemic health outcomes is essential.

This systematic review aimed to assess the effects of prosthodontic rehabilitation on nutritional and general health outcomes in elderly edentulous individuals and compare outcomes across prosthetic modalities. The objectives of this review are as follows:

1. Evaluate the effects of prosthodontic rehabilitation on nutritional outcomes (dietary intake, anthropometry, and biomarkers) in elderly edentulous populations.
2. Assess its impact on general health outcomes, including frailty, morbidity, and mortality.
3. Compare outcomes across different prosthetic interventions (conventional dentures, RPDs, implant‐supported overdentures, and fixed prostheses).

## 2. Methods

### 2.1. Protocol and Registration

This systematic review was conducted in accordance with the Preferred Reporting Items for Systematic Reviews and Meta‐Analyses (PRISMA) 2020 statement [18] and was prospectively registered in the PROSPERO database (registration number: CRD420251147518).

### 2.2. Eligibility Criteria

Eligibility was defined via the PICO framework:

• Population (P): Edentulous elderly individuals aged ≥ 60 years.

• Interventions (I): Prosthodontic rehabilitation, including CDs, RPDs, implant‐supported overdentures, single‐implant overdentures, and fixed prostheses.

• Comparators (C): Baseline (pretreatment), no intervention, or alternative prosthodontic modalities.

• Outcomes (O):

Primary outcomes: Nutritional outcomes, which include dietary intake, nutrient adequacy, malnutrition indices (e.g., MNA), anthropometry (BMI and weight), and biochemical markers (e.g., serum protein, albumin, calcium, and micronutrients). The general health outcomes included frailty, morbidity, hospitalization, and mortality.

Secondary outcomes: Patient‐centered measures, including OHRQoL and satisfaction.

• Study designs: RCTs, cohort, case–control, and before–after studies. Cross‐sectional observational studies reporting relevant outcomes were included to provide context.

• Language: Only English‐language, peer‐reviewed studies were considered.

Exclusion criteria: Articles that did not contribute original empirical data, such as review articles, conference abstracts, editorials, and simulation‐based studies, were excluded from this review. Additionally, studies that focused exclusively on prosthetic interventions for maxillofacial defects or rehabilitation following head and neck cancer treatment were also excluded, as they fell outside the scope of standard prosthodontic care being evaluated.

### 2.3. Information Sources

We searched PubMed/MEDLINE, Cochrane CENTRAL, and Scopus for eligible studies, with coverage from January 2000 up to September 2025. The reference lists of the included studies and relevant reviews were screened for additional publications. Only published full‐text articles were included.

### 2.4. Search Strategy

The search strategy involved the use of controlled vocabulary (MeSH) and free‐text terms with the Boolean “OR” and “AND” operators: edentulism, prosthodontics, CDs, overdentures, implants, nutrition, dietary intake, malnutrition, and elderly. Details of the search strategy are shown in Table 1.

**Table 1:** Search strategy and retrieval summary.

Database	Search period	Search string (simplified representation)	Filters applied	Records retrieved (*n*)
PubMed/MEDLINE	Jan 2000–Sept 2025	(“edentulous mouth”[MeSH] OR edentulism OR “tooth loss”) AND (“prosthodontics”[MeSH] OR “complete denture” OR overdenture OR “implant‐supported prosthesis”) AND (“nutrition”[MeSH] OR “dietary intake” OR “nutritional status” OR “malnutrition” OR “elderly”)	Article language: English	796
Cochrane CENTRAL	Jan 2000–Sept 2025	(edentulous OR edentulism OR “tooth loss”) AND (“complete denture” OR overdenture OR “implant‐supported prosthesis”) AND (nutrition OR “dietary intake” OR “quality of life”)	English, trials only	243
Scopus	Jan 2000–Sept 2025	TITLE‐ABS‐KEY (edentulous OR edentulism OR “tooth loss”) AND TITLE‐ABS‐KEY (“complete denture” OR overdenture OR “implant‐supported prosthesis”) AND TITLE‐ABS‐KEY (nutrition OR “dietary intake” OR “body mass index” OR frailty OR “quality of life”)	Article type: Journal, Language: English	449

### 2.5. Selection Process

A rigorous multistage screening process was implemented in accordance with the PRISMA 2020 statement [18]. All records retrieved from PubMed/MEDLINE, Cochrane CENTRAL, and Scopus were first imported into Covidence, where duplicates were automatically detected and subsequently verified manually by two reviewers. Title and abstract screening were then performed independently by two reviewers using predefined inclusion and exclusion criteria, with all decisions entered separately into Covidence. Any discrepancies were flagged automatically and resolved through consensus, and unresolved conflicts were adjudicated by a third senior reviewer. Full‐text articles of all potentially eligible studies were similarly screened independently by the same two reviewers, with reasons for exclusion documented for each study and reported in the PRISMA flow diagram. Reviewers worked independently at all stages and were blinded to each other’s decisions until submission, ensuring unbiased assessment. No automation, machine learning, or AI‐based tools were used for judgment or prioritization; Covidence functioned solely as a management platform for record organization, conflict flagging, and workflow tracking.

### 2.6. Data Extraction

Data were extracted independently by two reviewers using a predesigned, standardized extraction form to ensure consistency. Extracted information included study identifiers (author, year of publication, and country) and methodological characteristics such as study design. Population details captured age distribution and the number of participants. Intervention characteristics included the specific type of prosthodontic rehabilitation (CDs, RPDs, implant‐supported overdentures, single‐implant overdentures, and fixed prostheses), and these were documented alongside the comparator (baseline, no intervention, or alternative prosthodontic modalities). All reported nutritional, functional, and systemic outcomes were extracted, along with the duration of follow‐up. Finally, the key findings relevant to the review objectives were summarized. When information was unclear or missing—such as incomplete reporting of biochemical marker units, absence of exact numerical dietary intake values, or unspecified prosthetic materials—no assumptions or imputations were made. Instead, such variables were noted as “not reported” and interpreted qualitatively when possible. No attempts were made to estimate missing values or standardize outcomes across studies because of substantial variation in reporting formats. This approach ensured that all extracted data accurately reflected the original study reports without introducing interpretative bias.

### 2.7. Risk of Bias Assessment

For RCTs, methodological quality was assessed via the Cochrane Risk of Bias 2.0 tool (RoB‐2) [19] across five domains: randomization process, deviations from intended interventions, missing outcome data, measurement of outcomes, and selection of reported results. For observational studies, the Newcastle–Ottawa Scale (NOS) [20] was used, covering selection, comparability, and outcome domains. Judgments were summarized as low risk, some concerns/moderate risk, or high risk of bias.

### 2.8. Data Synthesis

A narrative synthesis was conducted as the studies differed widely in populations, interventions, and outcome measures. No statistical synthesis, conversions, imputations, or data transformations were performed because the outcome measures and reporting formats were not comparable across studies. The results are presented descriptively, supported by summary tables (selection process, study characteristics, and risk of bias) and figures (PRISMA diagram). Meta‐analysis was not performed, as studies are too heterogeneous to be comparable, as the meta‐analytical results may be meaningless, and true effects may be obscured.

## 3. Results

### 3.1. Study Selection

**Figure 1:**
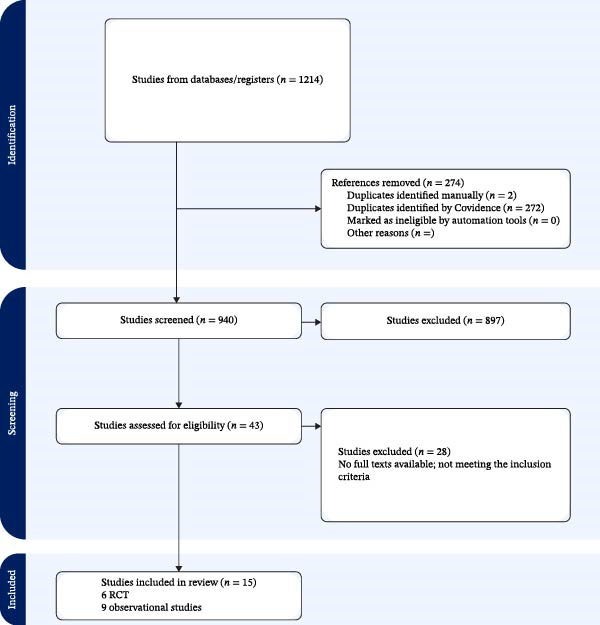
PRISMA 2020 flow diagram.

The database searches yielded 1214 records. After the removal of duplicates, 940 titles and abstracts were screened. Forty‐three full texts were assessed, 15 of which met the inclusion criteria. The reasons for exclusion were as follows: population not meeting the age criteria (<60 years), intervention not involving prosthodontic rehabilitation, outcomes not relevant to nutrition or general health, or insufficient outcome reporting. The selection process is depicted in the PRISMA 2020 flow diagram (Figure 1 and Table 1).

### 3.2. Study Characteristics

The 15 included studies included six RCTs [11–13, 21–23] and nine observational studies [10, 14, 15, 17, 24–28]. Studies were conducted across India, Japan, Brazil, Sweden, the UK/Ireland, Lebanon, Romania, and Serbia. Sample sizes ranged from *n* ≈ 60 in smaller RCTs to >300 in large observational studies. Follow‐up varied from immediate posttreatment to 6 months in most trials, whereas observational designs provided cross‐sectional insights.

#### 3.2.1. Interventions Evaluated

The interventions evaluated across the included studies encompassed a wide range of prosthodontic treatment modalities. These included conventional CDs, RPDs, and fixed partial dentures (FPDs), as well as implant‐retained overdentures, mini‐implant overdentures, and single‐implant overdentures. Several studies also assessed fixed implant prostheses. In addition, some trials implemented combined interventions in which prosthetic treatment was supplemented with individualized dietary counseling to enhance nutritional outcomes.

#### 3.2.2. Outcome Measures

The studies assessed multiple categories of outcomes. Anthropometric measures included body weight and BMI, while nutritional indices were evaluated using validated tools such as MNA [29] and SCREEN‐14 [30]. Dietary intake was measured through food frequency questionnaires, 24‐h dietary recalls, and nutrient adequacy scores. Biochemical markers such as serum albumin, calcium, total protein, and cholesterol were reported in several studies to quantify physiological changes after rehabilitation. Functional outcomes included bite force, masticatory efficiency, and other objectively measured indicators of oral function. Patient‐reported outcomes primarily focused on OHRQoL, assessed through instruments such as the OHIP [31] and GOHAI [32]. Table 2 provides the detailed characteristics and outcome domains of the included studies.

**Table 2:** Summary of study characteristics.

Reference	Country	Study design	Population (age, *n*)	Intervention	Comparator	Outcomes assessed	Follow‐up	Key findings
Ferreira de Sá et al. [21]	Brazil/multicentre	RCT/clinical (mandibular overdentures on 4 mini implants)	74 edentulous (mean age of 64.1 ± 8.0 years)	Mandibular overdentures retained by four mini‐implants (different implant distributions)	Different implant distributions (comparison within implant group)	Bite force (MBF), masticatory performance, OHIP‐Edent (OHRQoL), satisfaction	Posttreatment/short term	Implant distribution (larger implant area) improved functional parameters (MBF and MP); no clear effect on patient‐reported outcomes
Singh et al. [14]	India	Clinical (before–after observational clinical study)	100 complete‐denture wearers, geriatric (mean age 60 years)	New complete dentures	Baseline (preinsertion)	Serum calcium, total protein, phosphorus, hemoglobin	3 and 6 months	Improvements in serum calcium and protein at 3 months; mixed results for phosphorus/hemoglobin; overall positive effect on nutritional biomarkers
Aneja et al. [24]	India	Clinical (before–after observational clinical study)	250 geriatric patients (≥60 years)	CD, RPD, FPD, and implants (various)	Across modalities	BMI, macronutrients, iron (dietary), and anthropometry	5 months	Differences observed across modalities; conventional CDs often associated with larger anthropometric improvements vs. fixed prostheses in this sample
Vijaya et al. [12]	India	RCT (parallel and single‐blind)	68 geriatric patients (≥60 years)	New complete dentures + tailored progressive dietary advice	New complete dentures + standard care instructions	MNA, BMI, 3‐day 24‐h dietary recall (DietCal), and anthropometry	3 months	Intervention group (dietary advice + dentures) had significantly greater improvement in MNA and BMI vs. control; nutrient intake increases not statistically significant at 3 months
Homsi et al. [15]	Sweden	Case‒control	50 older adults (≥60 years)	Fixed implant prostheses	Natural dentate controls	MNA, SCREEN‐14, BMI, and dietary habits	None	Implant prosthesis group had higher BMI but mixed/poorer SCREEN‐14 and food variety findings
McKenna et al. [23]	UK/Ireland	Clinical/observational RCT‐related (impact of tooth replacement)	132 partially dentate older adults (≥65 years)	Tooth replacement/prosthodontic rehabilitation	Baseline/partially dentate comparisons	Masticatory performance and nutritional status (anthropometry/diet)	Posttreatment (short term)	Prosthodontic rehabilitation improved masticatory performance and was associated with better nutritional indicators; chewing performance predicted nutrition
Wallace et al. 2018 [22]	UK/Ireland	RCT	89 partially dentate older patients (≥65 years)	Prosthodontic rehabilitation (RCT intervention arm)	Control/baseline	Masticatory performance and nutritional outcomes	Posttreatment (short term)	Prosthodontic rehabilitation improved masticatory performance; masticatory gains associated with nutritional measures
Goel et al. [25]	India	Longitudinal (prospective)	135 older adults (≥60 years)	CD, RPD, and FPD (different prosthodontic modalities)	Across modalities	BMI, weight, protein, carbs, calories, iron, and vitamin B	6 months	Modality‐dependent improvements; conventional CDs showed notable improvements; CD > RPD > FPD in some nutritional measures
Sako et al. [13]	Japan	RCT (randomized controlled trial)	39 edentulous adults (mean age 73.4 ± 10.6 years)	Complete dentures + individualized dietary guidance	Complete dentures only	Nutritional biomarkers, dietary intake, occlusal force, and mastication	2–3 months	Increased fish intake and some biomarker improvements in intervention arm; occlusal force and mastication improved
Phanindra [26]	India	Clinical (before–after observational clinical study)	100 patients > 60 years (reported)	CD, RPD, and fixed prostheses	Across modalities	BMI, protein, carbs, iron, albumin, and cholesterol	5 months (reporting timeframe)	CD group showed greater short‐term improvements versus fixed prosthesis group in some nutritional indices
do Amaral et al. [27]	Brazil	Clinical (single‐implant overdenture wearers)	Older adults with single‐implant overdentures (mean age, 68.6 ± 5.2 years)	Single‐implant overdentures	Conventional dentures (or before)	Sensorial ability, mastication, and nutrition (anthropometry/diet)	Posttreatment	Single‐implant overdenture wearers showed improved sensorial ability, mastication, and some nutritional indicators vs. conventional denture wearers
Tanasić et al. [17]	Serbia	Cross‐sectional	200 Serbian elders (mean age, 68.9 ± 2.29 years)	Dentition status assessed (natural teeth, partial, and edentulous)	Comparisons by dentition status	Malnutrition risk (e.g., MNA)	None	Presence of poorer dentition/prosthetic problems associated with higher malnutrition risk
Amagai et al. [11]	Japan	RCT	62 edentulous individuals (age range 75–85 years)	Prosthetic rehabilitation + simple dietary counseling	Prosthetic rehabilitation only or baseline	Food intake and OHRQoL	6 months	Combined prosthetic rehab + simple dietary advice improved food intake and OHRQoL vs. prosthesis alone
El Osta et al. [28]	Lebanon/multicentre	Clinical/observational	Edentulous patients receiving implant‐supported prostheses (≥60 years)	Implant‐supported prostheses	Conventional dentures or baseline	Nutritional status and oral health perception	Short term	Implant‐supported prostheses had positive impact on patient‐reported oral health perception and some nutritional measures
Bida et al. [10]	Romania	Observational (cross‐sectional)	338 RPD wearers, mean age 64.09 ± 7.38 years)	RPD (metal framework ± interim acrylic)	Algorithm‐followed vs. nonalgorithm groups	OHRQoL (OHIP‐5)	None	Statistically significant differences in OHIP domains across groups; following the RPD algorithm associated with better OHRQoL

### 3.3. Risk of Bias

RCTs (*n* = 6): Most reported adequate randomization, but allocation concealment was not feasible. Blinding was generally not feasible for participants or outcome assessors. Four RCTs were judged to have “low risk,” and two had “some concerns.” Observational studies (*n* = 9): Most were rated at moderate risk of bias, reflecting nonrandom sampling, potential confounding, and reliance on self‐reported dietary intake. Two were classified as “low” risk. Table 3 (RoB‐2 for RCTs) and Table 4 (NOS for observational studies) provide detailed assessments.

**Table 3:** Risk of bias for RCTs (Cochrane RoB 2.0 tool).

Study	Randomization	Allocation concealment	Blinding	Incomplete data	Selective reporting	Overall risk
Ferreira de Sá et al. [21]	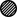 +	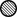 –	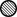 –	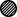 +	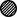 +	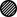 +
Vijaya et al. [12]	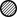 ?	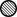 –	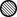 –	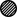 +	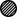 +	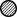 ?
McKenna et al. [23]	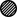 +	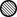 –	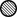 –	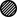 +	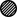 +	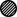 +
Wallace et al. [22]	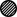 +	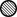 –	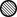 –	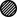 +	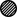 +	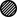 +
Sako et al. [13]	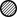 ?	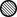 –	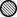 –	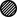 +	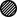 +	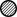 ?
Amagai et al. [11]	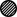 +	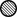 –	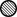 –	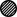 +	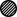 +	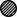 +

*Note:* 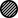 “+” Low risk, 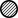 “?” some concerns, 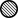 “–” not feasible for intervention.

**Table 4:** Risk of bias for observational studies (Newcastle–Ottawa Scale).

Study	Selection (max 4)	Comparability (max 2)	Outcome (max 3)	Total (max 9)	Risk of bias
Singh et al. [14]	3	1	1	5	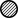
Aneja et al. [24]	3	1	2	6	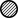
Homsi et al. [15]	3	1	3	7	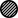
Goel et al. [25]	3	1	2	6	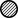
Phanindra [26]	3	1	2	6	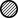
do Amaral et al. [27]	3	1	2	6	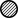
Tanasić et al. [17]	3	1	2	6	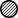
El Osta et al. [28]	3	1	2	6	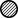
Bida et al. [10]	3	1	3	7	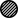

*Note:* 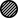 Low risk, 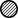 moderate risk.

### 3.4. Results of Individual Studies

Nutritional status (BMI and MNA): Two RCTs [12, 13] reported significant improvements in BMI and MNA when dietary counseling was provided alongside dentures. A clinical study [14] reported increases in serum calcium and protein but mixed results for phosphorus and hemoglobin. One RCT [11] reported improved food intake and OHRQoL with combined prosthetic rehabilitation and dietary advice. Observational studies [24–26] reported modality‐dependent improvements, with CDs often showing better short‐term gains than fixed prostheses.

Dietary intake: Increased protein, calorie, and fish/vegetable consumption was observed in RCTs that combined dietary guidance with prosthesis provision [12, 13]. Implant‐supported prostheses facilitate greater food variety [15, 28].

Biochemical markers: Evidence related to biochemical markers was limited. Two studies [13, 14] reported small favorable changes in the serum ALB and calcium levels. Other studies did not find consistent improvements.

Masticatory performance: Strong and consistent improvements in bite force and chewing efficiency were observed in implant‐supported overdenture trials [21, 27] and RCTs in partially dentate populations [22, 23].

OHRQoL: Improvements in OHRQoL were consistently reported across both RCTs and observational studies [10–12, 21]. Participants receiving new or upgraded prosthetic interventions especially implant‐supported options showed significant gains in comfort, chewing ability, social confidence, and overall satisfaction. These findings demonstrate that prosthodontic rehabilitation reliably enhances patient‐perceived oral health and daily functioning.

General health outcomes: A clinical study [17] revealed associations between poor dentition and increased malnutrition risk in Serbian elderly individuals. No included studies reported hospitalization or healthcare utilization outcomes. Mortality was not assessed in the final included studies.

### 3.5. Synthesis of Results

BMI and anthropometry: Most studies [12, 15, 24–26] reported modest yet consistent increases in BMI and other anthropometric indicators following prosthodontic rehabilitation. These improvements tended to be more pronounced when dietary counseling was incorporated, suggesting that functional gains alone may not sufficiently change nutritional status without accompanying behavioral guidance.

Dietary intake: Enhancements in dietary intake were observed across several studies [12, 13, 15, 28]. Participants with implant‐supported prostheses and those who received combined interventions (prosthesis plus counseling) demonstrated greater improvements in macronutrient adequacy, food variety, and the consumption of harder‐to‐chew foods such as fruits, vegetables, and protein‐rich items.

Biomarkers: Findings related to biochemical markers were mixed. While a few studies [14, 24] documented improvements in serum albumin, calcium, or protein levels, the evidence overall was inconsistent and insufficient to draw firm conclusions. Variability in laboratory methods and limited follow‐up periods likely contributed to these inconsistent results.

Masticatory performance and OHRQoL: Strong and consistent improvements were observed in masticatory performance [21–23, 27] and OHRQoL [10–12, 21] across all prosthetic modalities. Implant‐based interventions showed the most substantial benefits, with significant increases in bite force, chewing efficiency, and self‐reported satisfaction.

General health (frailty and morbidity): Evidence regarding general health outcomes such as frailty, morbidity, and mortality was sparse. Only one cross‐sectional study [17] demonstrated an association between poor dentition and increased malnutrition risk. No longitudinal data were available to establish a causal relationship between prosthodontic rehabilitation and broader systemic health outcomes.

### 3.6. Additional Analyses

Subgroup comparisons: CDs vs. implants: Implants provided superior functional outcomes but did not consistently yield better biochemical nutrition indices in the short term. Dietary counseling strongly influences outcomes, with combined interventions outperforming prosthesis‐only care.

Sensitivity analysis: This analysis was not performed because of the absence of pooled quantitative data.

#### 3.6.1. Meta‐Analysis Feasibility

Outcome heterogeneity, variation in measurement tools, and incomplete reporting of mean values and standard deviations precluded meta‐analysis.

## 4. Discussion

### 4.1. Principal Findings

This systematic review, which included 15 studies from various clinical and geographic contexts, revealed that prosthodontic rehabilitation regularly improves older edentulous people’s masticatory function [21–23, 27] and OHRQoL [10–12, 21].

The evidence for nutritional improvements is weaker but still suggestive; multiple trials reported moderate but significant increases in dietary intake, BMI, and MNA scores, especially when organized dietary coaching was provided in conjunction with prosthetic treatment [12, 13, 15, 28].

Although prosthodontic rehabilitation improves chewing ability and meal diversity, it is not enough to address malnutrition in older denture wearers. Many edentulous patients rely on soft, carbohydrate‐rich diets because they have trouble chewing or are afraid of food impaction, which causes them to avoid fibrous fruits, vegetables, and protein sources. This puts one at risk for deficiencies in minerals (calcium, iron, and zinc) and vitamins (A, B12, C, D, and folate), which present as anemia, mucosal fragility, mouth lesions, and osteoporosis. Nutritional susceptibility, including decreased muscle strength, hyposalivation, and adverse drug reactions, is further exacerbated by physiological aging. Consequently, nutritional analysis and counseling should be combined with prosthetic treatment, ideally incorporated into the denture construction process. Personalized dietary recommendations should be given on recall visits, and nutritional assessment instruments (such as the MNA) should be used consistently [16, 33].

Better bite force, chewing efficiency, and meal variety are all offered by overdentures and implant‐retained prostheses [21–23, 27]; however, these functional advantages do not always translate into measurable increases in biochemical markers or systemic health outcomes such as mortality, morbidity, or frailty.

The discrepancy between mild systemic alterations and large functional gains can be explained by biological and temporal considerations. Immediate occlusion restoration enhances chewing skills and increases meal variety. However, prolonged dietary alteration, sufficient nutrient absorption, and longer follow‐up, typically ≤6 months, are necessary for quantifying changes in nutritional biomarkers and systemic endpoints [33, 34].

Two studies showed that gains in nutritional indices were more reliable when dietary guidance was incorporated into prosthetic treatment [12, 13]. This implies that nutritional counseling serves as the behavioral catalyst needed to convert functional gains into quantifiable dietary changes, whereas prosthetic rehabilitation serves as a necessary but insufficient prerequisite for systemic health benefits.

Owing to their biomechanical advantages, implant‐supported prostheses are often considered superior to conventional dentures. This functional advantage is supported by our review, which revealed improved masticatory efficiency and bite force [21, 27].

However, the lack of persistent superiority in systemic markers emphasizes how the link between nutrition and oral function is mediated by social context, dietary practices, and patient adaptation. This subtlety calls into question the notion that implants by themselves will always result in improved nutritional outcomes, highlighting the importance of comprehensive, multidisciplinary care.

### 4.2. Clinical Implications

The implications for prosthodontists are as follows: incorporating functional prostheses with dietary counseling optimizes health benefits while restoring masticatory competence. To ensure that patients not only regain the ability to chew food but also adopt and maintain diets high in protein, fiber, and micronutrients, routine practice should consider working in conjunction with nutritionists and geriatric care providers. These results indicate that the nutritional status of dieticians and geriatric doctors is influenced by oral health. Malnutrition and frailty assessments in senior care should include screening for dental status and prosthesis appropriateness.

### 4.3. Public Health Relevance

Prosthodontic rehabilitation should be reframed as a public health intervention with systemic and nutritional implications rather than only as a restorative dental procedure at the community level. Integrated programs that combine prosthodontic services with community‐based nutritional counseling could be crucial in lowering frailty and enhancing quality of life in low‐ and middle‐income countries such as India, where geriatric malnutrition and edentulism are both highly prevalent.

The incorporation of such approaches into national oral health and elderly care policies may yield both clinical and economic benefits through reduced hospitalizations and improved functional independence.

### 4.4. Strengths and Limitations

This review’s strengths include a thorough multidatabase search, the inclusion of both randomized and observational evidence, dual‐reviewer screening and extraction, and a structured risk‐of‐bias evaluation using well‐known methodologies (RoB‐2 and NOS). The synthesis also encompassed a wide range of outcomes, spanning functional, nutritional, and systemic domains, providing a holistic picture of prosthodontic rehabilitation in elderly individuals.

This study has several limitations. First, the majority of studies had small sample sizes and brief follow‐up periods, which made it difficult to record systemic outcomes such as death or frailty. Second, formal meta‐analysis was not possible due to outcome heterogeneity, which included anything from biochemical testing to self‐reported dietary intake. Third, recollection and reporting bias are introduced by the fact that many studies rely on self‐reported dietary measurements. Fourth, causal inference is hindered by a lack of adjustment for confounders such as comorbidity, socioeconomic level, and physical function. Finally, restricting inclusion to English‐language publications may have excluded relevant evidence.

### 4.5. Future Research Directions

Future research should perform extensive, multicenter RCTs that compare prosthetic modalities with and without organized dietary interventions via standardized interventions and outcome measurements. They should use biochemical indicators (such as albumin, vitamin D, and iron) and establish nutritional indices as the main objectives and increase follow‐up to evaluate the effects on mortality, hospitalization rates, and frailty trajectories.

## 5. Conclusion

Prosthodontic rehabilitation in older adults reliably restores chewing function and enhances OHRQoL, with modest but consistent improvements in nutritional outcomes, particularly when combined with dietary counseling. However, evidence for long‐term systemic benefits such as reduced frailty, morbidity, or mortality remains limited. These findings highlight the need for integrated clinical pathways linking prosthodontic care with nutritional and geriatric services and for robust multicenter trials to establish the broader health and economic value of prosthetic rehabilitation in aging populations.

## Funding

The authors declare that no financial support was received for the research and/or publication of this article. This work did not receive any specific grant from funding agencies in the public, commercial, or not‐for‐profit sectors.

## Disclosure

All content generated or refined with the assistance of ChatGPT was critically reviewed, verified, and approved by the authors, who take full responsibility for the integrity, accuracy, and originality of the manuscript.

## Conflicts of Interest

The authors declare no conflicts of interest.

## Data Availability

The datasets used during the current study are available from the corresponding author upon reasonable request.
